# Studies of Sol-Gel Evolution and Distribution of Eu^3+^ Ions in Glass–Ceramics Containing LaF_3_ Nanocrystals Depending on Initial Sols Composition

**DOI:** 10.3390/ijms22030996

**Published:** 2021-01-20

**Authors:** Natalia Pawlik, Barbara Szpikowska-Sroka, Tomasz Goryczka, Wojciech A. Pisarski

**Affiliations:** 1 Institute of Chemistry, University of Silesia, 40-007 Katowice, Poland; barbara.szpikowska-sroka@us.edu.pl; 2 Institute of Materials Engineering, University of Silesia, 41-500 Chorzów, Poland; tomasz.goryczka@us.edu.pl

**Keywords:** oxyfluoride glass–ceramics, sol-gel method, Eu^3+^ ions, fluoride nanocrystals

## Abstract

In this work, we performed a systematic analysis of the impact of selected chemical reagents used in sol-gel synthesis (i.e., N,N-dimethylformamide) and different catalyst agents (i.e., CH_3_COOH, HNO_3_) on the formation and luminescence of Eu^3+^-doped SiO_2_–LaF_3_ nano-glass–ceramics. Due to the characteristic nature of intra-configurational electronic transitions of Eu^3+^ ions within the 4f^6^ manifold (^5^D_0_ → ^7^F_J_, J = 0–4), they are frequently used as a spectral probe. Thus, the changes in the photoluminescence profile of Eu^3+^ ions could identify the general tendency of rare earth materials to segregate inside low-phonon energy fluoride nanocrystals, which allows us to assess their application potential in optoelectronics. Fabricated sol-gel materials, from sols to gels and xerogels to nano-glass–ceramics, were examined using several experimental techniques: X-ray diffraction (XRD), transmission electron microscopy (TEM), infrared spectroscopy (IR), and luminescence measurements. It was found that the distribution of Eu^3+^ ions between the amorphous silicate sol-gel host and LaF_3_ nanocrystals is strictly dependent on the initial composition of the obtained sols, and the lack of N,N-dimethylformamide significantly promotes the segregation of Eu^3+^ ions inside LaF_3_ nanocrystals. As a result, we detected long-lived luminescence from the ^5^D_0_ excited state equal to 6.21 ms, which predisposes the obtained glass–ceramic material for use as an optical element in reddish-orange emitting devices.

## 1. Introduction

Nowadays, rare earth-doped materials are widely dedicated to fulfilling many sophisticated and active functions in commercial products due to their interesting and unique optical, electrical, and magnetic properties. These materials are being utilized as temperature imaging systems, e.g., Sr_4_Al_14_O_25_:Mn^4+^, Tb^3+^ nanocrystals [1], or Ba_2_TiGe_2_O_8_:Ln (Ln = Eu^3+^, Er^3+^, Ho^3+^, Yb^3+^) phosphors [2], and in biological systems for in vitro/in vivo imaging analyses, e.g., Y_2_O_3_:Nd^3+^, Er^3+^ phosphors [3]. Thin films based on selected rare earth titanates (e.g., Sm-doped EuTiO_3_, SmTiO_3_/SrTiO_3_ interface) are considered reliable in the development of topological antiferromagnetic spintronics, the examination of novel superconducting states, or field tunable devices [4,5,6]. Therefore, rare earth-doped materials have many technological advantages, i.e., reduced energy consumption and greater working efficiency; hence, they have become critical to modern technologies ranging from light emitting diodes (LED) [7] to wind turbines [8].

Oxyfluoride nano-glass–ceramics doped with optically active rare earth ions (RE^3+^) have gained great attention due to their favorable behavior as a result of mixing the advantages of fluoride nanocrystals with an oxide glassy host [9,10,11,12,13]. Indeed, because of their undoubtedly outstanding properties, such a class of optical materials is indispensable in the development of optoelectronics, offering promising applications in optical thermometers [14,15,16,17], LEDs [18,19,20], waveguides [21], and lasers [22]. The optical properties of oxyfluoride nano-glass–ceramics depend on several fundamental factors. Firstly, an enhancement of luminescence originating from RE^3+^ dopants is strongly determined by phonon energy in the adjacent vicinity around them: for fluorides, the energies are in a range from 250 cm^−1^ (e.g., for β-PbF_2_ phase [23]) to almost 600 cm^−1^ (e.g., for LiYF_4_ or LiGdF_4_ [24,25]), which are significantly lower compared with glassy oxide matrices, like silicate (~1100 cm^−1^ [26]), phosphate (~1250 cm^−1^ [27]), or borate (~1350 cm^−1^ [28]). In other words, the segregation of RE^3+^ ions inside fluoride nanocrystals with low phonon energy successfully suppresses the undesirable non-radiative losses and achieves higher luminescence efficiencies. Nonetheless, in this class of materials, the choice of a suitable crystal lattice is only one of the preconditions determining the enhancement of RE^3+^ luminescence. It should be noted that a crucial factor lies in the tendency of RE^3+^ ions to migrate from the oxide glassy host into fluoride nanocrystals during controlled heat treatment [29,30,31]. Another factor which strongly determines the optical properties of nano-glass–ceramics is the precise control of nanocrystal size. To avoid Rayleigh scattering, the size of fluoride nanocrystals should not exceed 40–50 nm (due to considerable differences in the refractive indexes of the glassy oxide host and the fluoride crystal phase) [32].

The majority of the oxyfluoride nano-glass–ceramics characterized and described in the literature were prepared by the conventional melt–quenching method, followed by heat treatment at a controlled and specified time and temperature [33,34,35,36,37,38,39,40]. However, despite many undoubted advantages, the melt–quenching method has substantial drawbacks related to the risk of the evaporation of more volatile compounds during glass melting at temperatures exceeding 1000 °C. In fact, these melting conditions cause actual losses of fluorides, limiting the amounts of the crystal phase in fabricated glass–ceramic materials [9]. To overcome this important limitation, the sol-gel method is used as an alternative synthesis route because the appropriate chemical reactions (i.e., hydrolysis of the metal/semi-metal alkoxide, further condensation, and polycondensation) are usually performed at room temperature (RT) [41,42]. As a result, this low temperature approach allows for the fabrication of nano-glass–ceramics with a greater fluoride crystal fraction. Additionally, sol-gel processing offers a high degree of compositional and homogeneity control inherent with the synthesis being performed in a liquid phase, ensuring easy fabrication in comparison with the conventional melt–quenching method, in which phase separation in glass could be observed (related to the immiscibility of fluorine in a glassy oxide host) [43,44,45].

A crucial aspect of determining the utility potential of oxyfluoride glass–ceramics concerns the enrichment of fluoride nanocrystals by RE^3+^ dopant ions during the controlled heat treatment of precursor xerogels. Since the sol-gel hosts are dynamic at every stage of synthesis, Secu et al. [46] examined the photoluminescence behavior of Eu^3+^ in sols, through xerogels and up to SiO_2_–BaF_2_ nano-glass–ceramics, and the impact of heat treatment temperature (320 °C, 540 °C, and 800 °C) on the migration tendency of Eu^3+^ ions into BaF_2_ nanocrystals. Based on changes in red-to-orange ratio values, R/O, and photoluminescence decay analysis, it was proven that Eu^3+^ ions were segregated more preferably in BaF_2_ nanocrystals at 800 °C, characterized by their slightly larger size (7 nm) compared to that formed at lower treatment temperatures (3–4 nm at 540 °C). According to the specific spectral behavior of Eu^3+^ ions, Velázquez et al. [47] stated that for 90SiO_2_–10GdF_3_ and 80SiO_2_–20GdF_3_ nano-glass–ceramics, Eu^3+^ ions were preferably embedded inside GdF_3_ nanocrystals, which was also confirmed by results from energy dispersive X-ray (EDX) analysis. Kawamura et al. [48] studied the distribution of Nd^3+^ ions inside silicate nano-glass–ceramics containing LiYF_4_ and/or YF_3_ nanocrystals and the influence of selected factors like treatment temperature (500 °C, 600 °C) and the molar ratio of Li(CH_3_COO):Y(CH_3_COO)_3_:TEOS (tetraethoxysilane) on the enrichment of fluoride nanocrystals by a Nd^3+^ dopant. For this purpose, bright-field transmission electron microscopy (BF TEM) and EDX mapping were performed. The tendency for the predominant incorporation of Nd^3+^ ions into fluoride nanocrystals was verified for all samples, regardless of treatment temperature and Li:Y:Si molar ratio. Gorni et al. [49] studied the segregation of Er^3+^ ions inside LaF_3_ nanocrystals in 80SiO_2_–20LaF_3_:Er^3+^ thin films and self-supported layers based on TEOS and TEOS–MTES (methyl-trimethoxysilane). Based on X-ray absorption near edge structure (XANES) spectra and electron paramagnetic resonance (EPR) techniques, it was found that Er^3+^ ions were likely situated in LaF_3_ fluoride nanocrystals in the fabricated samples. Moreover, the authors performed a series of luminescence measurements at low temperature (T = 9K), which also confirmed the incorporation of Er^3+^ in LaF_3_ due to the well-resolved structure of the recorded bands. However, some differences in the PL spectra for thin films and bulk samples were found, which was related to the distribution of Er^3+^ between sol-gel hosts and fluoride nanocrystals. Therefore, the change in qualitative and/or quantitative composition of sol-gel materials could significantly modulate their final structural properties, and consequently determine the luminescence behavior of RE^3+^. According to our previous results for oxyfluoride Eu^3+^-doped nano-glass–ceramics containing MF_3_ (M = La, Y, Gd) nanocrystals [50,51,52], the most efficient segregation of Eu^3+^ inside the fluoride crystal lattice was reported for the SiO_2_–LaF_3_ system. Since the phonon energies of individual MF_3_ crystal lattices are nearly the same (LaF_3_: 350 cm^−1^ [53], YF_3_: 358 cm^−1^ [54], GdF_3_: 360 cm^−1^ [55]), the probable reason for the increased tendency of Eu^3+^ migration into the LaF_3_ hexagonal crystal system is the higher coordination number (CN = 11) [56], compared with YF_3_ and GdF_3_ crystallized phases in the orthorhombic crystal system (CN = 9) [57,58]. Thus, we have chosen the SiO_2_–LaF_3_:Eu^3+^ nano-glass–ceramic system to study the impact of selected chemical components on the distribution of Eu^3+^ ions between LaF_3_ nanocrystals and the silicate sol-gel host.

In this work, we performed a systematic analysis of the influence of different initial compositions on the formation and luminescence of oxyfluoride Eu^3+^-doped SiO_2_–LaF_3_ nano-glass–ceramics. In this case, we discussed the role of N,N-dimethylformamide (commonly used as a drying-control chemical additive (DCCA) agent [59]), and select catalyst agents (acetic acid, nitric acid) on the partition of Eu^3+^ into low phonon energy LaF_3_ nanocrystals during controlled ceramization. The impact of N,N-dimethylformamide (DMF) and catalysts on sol-gel transformation was reported and investigated in detail using the XRD technique, TEM microscopy, IR spectroscopy, and luminescence measurements. It can be clearly stated that the segregation of Eu^3+^ ions inside LaF_3_ nanocrystals is strongly dependent on the initial composition of fabricated sols.

## 2. Results and Discussion

### 2.1. The Impact of Initial Composition on the Sol-Gel Transformation of Liquid Sols into Xerogels

**Figure 1:**
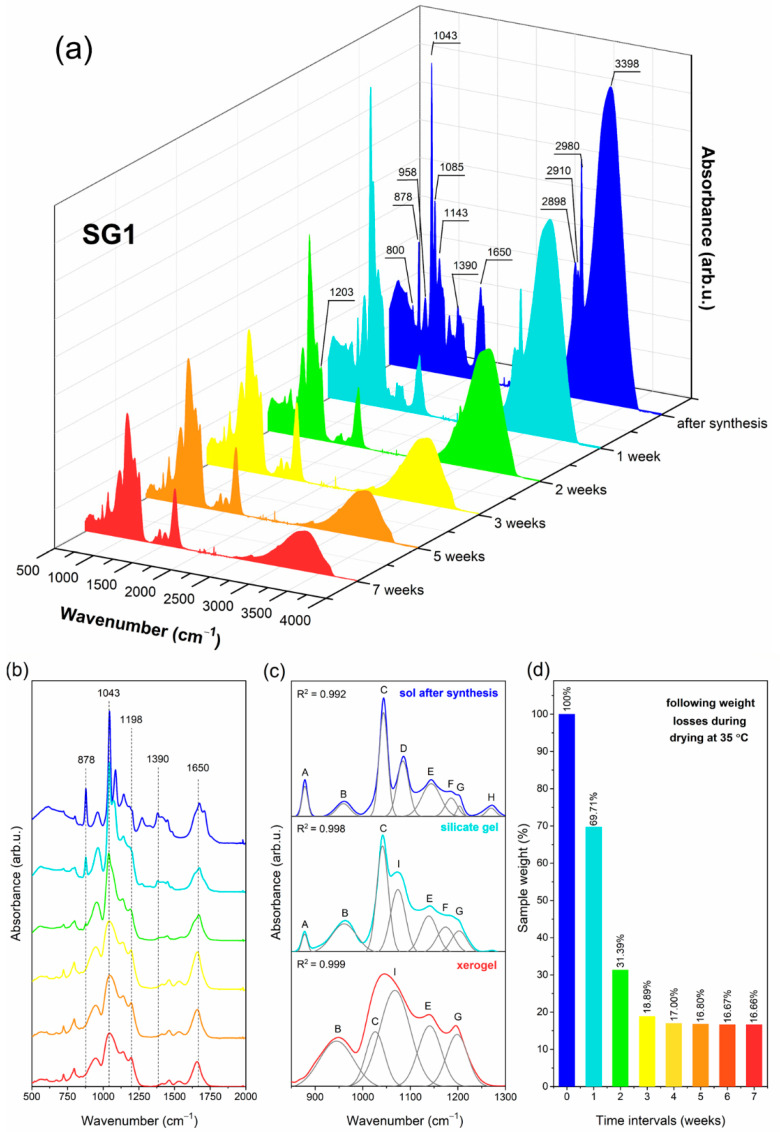
IR spectra recorded at various time intervals for samples with nominal composition SG1 (**a**), the enlargement of the 500 cm^−1^–2000 cm^−1^ frequency region (**b**), deconvolution within the 850 cm^−1^–1300 cm^−1^ frequency region (**c**), and weight losses identified during drying at 35 °C at specific time intervals (**d**).

**Figure 2:**
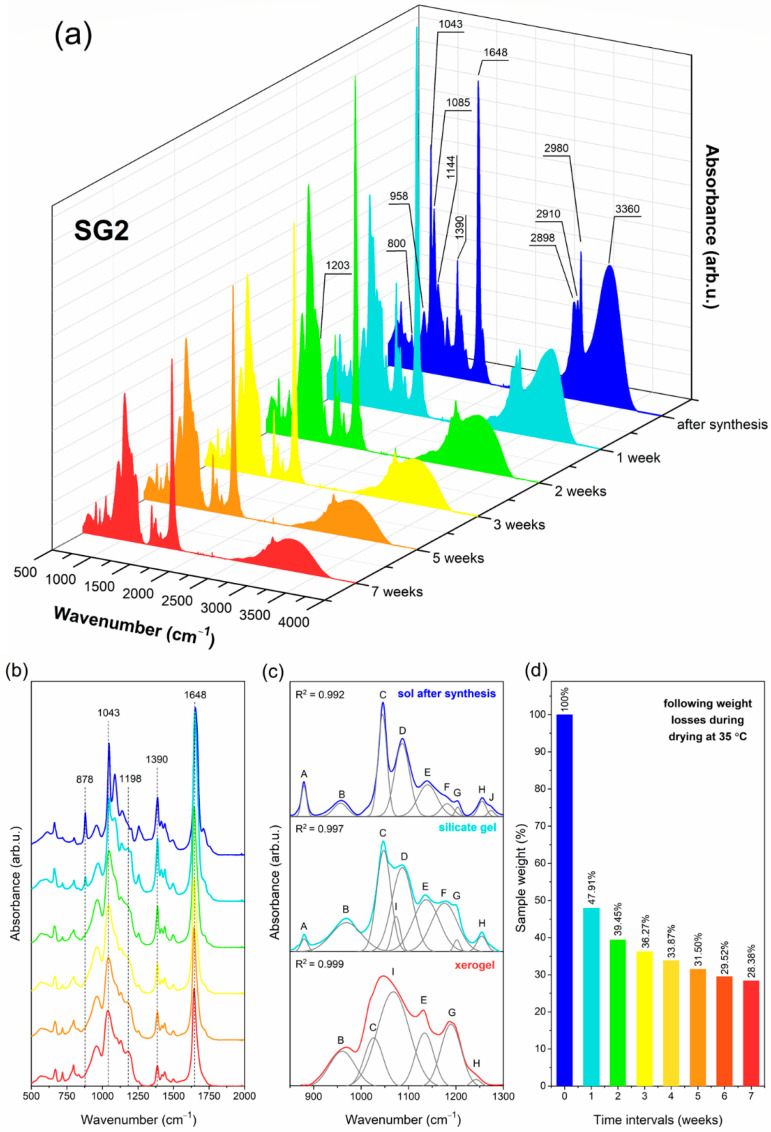
IR spectra recorded at various time intervals for samples with nominal composition SG2 (**a**), the enlargement of the 500 cm^−1^–2000 cm^−1^ frequency region (**b**), deconvolution within the 850 cm^−1^–1300 cm^−1^ frequency region (**c**), and weight losses identified during drying at 35 °C at specific time intervals (**d**).

**Figure 3:**
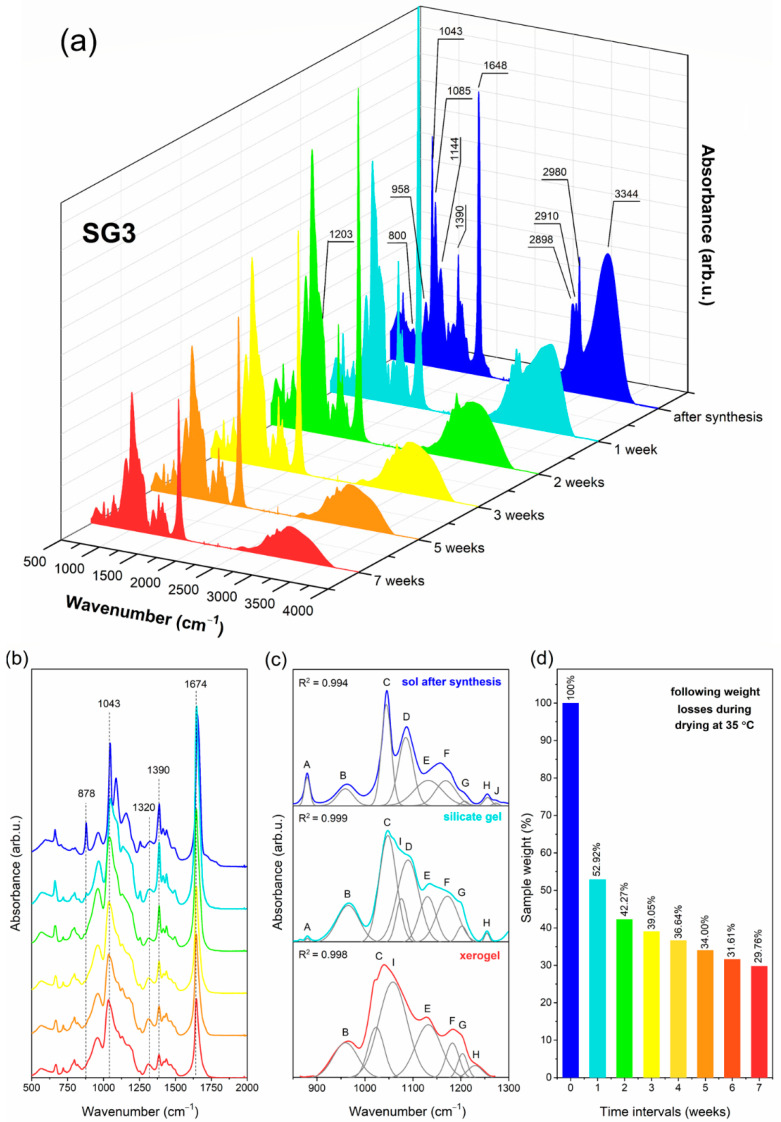
IR spectra recorded at various time intervals for samples with nominal composition SG3 (**a**), the enlargement of the 500 cm^−1^–2000 cm^−1^ frequency region (**b**), deconvolution within the 850 cm^−1^–1300 cm^−1^ frequency region (**c**), and weight losses identified during drying at 35 °C at specific time intervals (**d**).

Prepared sol-gel materials are dynamic from the initial processing stages to the glass-ceramics, including gels and xerogels. Indeed, the microstructure of these materials undergoes a continuous rearrangement according to the creation of a polycondensed silicate network and the gradual evaporation of the used solvents. The IR technique is a valuable tool for identifying the specific evolution of these materials closely related to sol-gel processing peculiarities. To determine the impact of the DMF additive and modification to the catalyst agent on creating a sol-gel network, the IR spectra were recorded in the 500 cm^−1^–4000 cm^−1^ frequency region in selected time intervals and the recorded signals were compared with literature data [60,61,62,63]. The evolution of infrared signals for each sample from sols up to xerogels is depicted in Figure 1 (SG1), Figure 2 (SG2), and Figure 3 (SG3). To better observe the changes in the shape of the recorded IR spectra, the enlargement of the 500 cm^−1^–2000 cm^−1^ region is shown. Moreover, for a more in-depth interpretation of oscillations within the 850 cm^−1^–1300 cm^−1^ region, deconvolution was performed. Peak fitting during deconvolution was done using a Gauss function with a squared regression coefficient of R^2^ ≥ 0.992 (Figure 1, Figure 2 and Figure 3). Simultaneously, the weight loss investigations at appropriate time intervals were carried out, and the results are also presented. For better readability, a detailed list of wavenumbers for individual infrared signals is depicted in Table 1.

The IR spectra recorded for all liquid sols (SG1–SG3) obtained directly after sol-gel synthesis clearly revealed the impressive amounts of water and organic solvents used: ethyl alcohol, acetic acid, DMF, and residues of unreacted TFA. This can be seen from the intense and broad band with a maximum at 3230 cm^−1^, as well as other infrared signals which could be assigned to C–H vibrations (1390 cm^−1^, 1460 cm^−1^, 2898 cm^−1^, 2910 cm^−1^, 2980 cm^−1^) and C=O groups (1648 cm^−1^, 1650 cm^−1^, 1712 cm^−1^). It should also be noted that the relative intensities of the broad OH band and sharp C–H signals for SG1 are greater that those for SG2 and SG3, which is caused by the larger amount of water and ethyl alcohol used during synthesis (indeed, the molar ratio of TEOS:EtOH:H_2_O is equal to 1:4:10 for SG1, and 1:2:4 for SG2 and SG3). Simultaneously, the intensity of the signal near 1648 cm^−1^/1650 cm^−1^ (C=O group vibration) is impressively high for SG2 and SG3 compared to SG1, which shows a clear contribution of the DMF additive on the formation of this band. Moreover, it is interesting to note that for SG2 and SG3, the signal near 2812 cm^−1^ (C–N bond vibrations) is hidden by a broad OH band; hence, it is not visible at this stage of sol-gel transformation.

The spectra recorded for the initial sols also reveal that hydrolysis and polycondensation reactions had already begun, and the silicate network began to form. However, the occurrence of an infrared signal at 1086 cm^−1^ (band D) assigned to vibrations inside Si–O–C groups indicates incomplete hydrolysis for all obtained sols. Nevertheless, deconvolution within the 850 cm^−1^–1300 cm^−1^ region revealed the creation of the first cyclic, two-fold siloxane rings, Q^2^ ((Si(O_1/2_)_2_O_2_)^−2^), Q^3^ ((Si(O_1/2_)_3_O)^−^) and Q^4^ units (Si(O_1/2_)_4_) at 878 cm^−1^ (band A), 958 cm^−1^ (band B), 1043 cm^−1^ (band C) and 1144 cm^−1^ (band E), respectively. “O_1/2_” corresponds to each oxygen atom that is involved in the formation of an Si–O–Si bridge; however, “O” is a non-bridging oxygen atom, and therefore the higher the value of the “n” index, the fewer Si–OH unreacted groups are present. For all fabricated sols, the deconvoluted component at 1043 cm^−1^ is the most intense, which could suggest that the amounts of Q^3^ units are greater than Q^n^ (n = 2, 4) species. On the other hand, the bands at 1144 cm^−1^ and 1203 cm^−1^ can also be interpreted as vibrations originating from a C–F bond inside the CF_3_ groups in trifluoroacetates. Moreover, according to transverse–optical (TO) and longitudinal–optic modes (LO), the formation of Si–O–Si siloxane bridges was confirmed. For all sols, LO_4_ (1186 cm^−1^) and TO_4_ (1203 cm^−1^) modes were recorded. For SG2 and SG3, an infrared signal near 1255 cm^−1^ (band H) was also identified, which could be assigned to the LO_3_ mode according to the literature data. However, this band could not be univocally assigned to such a mode as it may also point N–C bond vibration within DMF molecules. The last deconvoluted component recorded at 1274 cm^−1^ (band J) could be assigned to the –CH_2_CH_3_ group of ethyl alcohol.

After one week of drying at 35 °C, the sols were converted into liquid-filled porous wet gels. This step is correlated with intense evaporation of water and ethyl alcohol from the dynamic microporous sol-gel structures. The intensity of the broad band recorded in the frequency region >3000 cm^−1^ began to decrease, similarly to the adjacent signals originating from C–H bonds (2898 cm^−1^, 2910 cm^−1^, 2980 cm^−1^). Interestingly, for the SG1 sample, the second maximum located at 3398 cm^−1^ of the broad band starts to appear, corresponding to hydrogen-bonded Si–OH groups. Nevertheless, the amounts of water and ethyl alcohol were still considerable. Indeed, these compounds could be successfully “trapped” inside the forming silicate sol-gel network due to hydrogen bonding with unreacted Si–OH groups. It was verified that the remaining gel masses were 69.71%, 47.91% and 52.92% of the initial weights of the SG1, SG2, and SG3 samples, respectively. The relatively smaller weight loss identified for the SG1 gel may result from the presence of the greatest amounts of Si–OH groups formed at this stage of sol-gel evolution (a band recorded above the 3000 cm^−1^ frequency region with visible maxima at 3398 cm^−1^ and 3230 cm^−1^ is the most intense compared to the SG2 and SG3 gels). Indeed, such Si–OH groups are able to effectively hydrogen-bond water and ethanol despite their introduction during sol-gel synthesis (the molar ratio of TEOS:EtOH:H_2_O is 1:4:10 for SG1, and the molar ratio of TEOS:EtOH:DMF:H_2_O is 1:2:2:4 for SG2 and SG3).

The deconvolution within the 850 cm^−1^–1300 cm^−1^ frequency range revealed the presence of a 878 cm^−1^ signal for all obtained gels (band A); however, its intensity is lower than that of the initial liquid sols, especially for SG3. Hence, it could be assumed that the two-fold silicate rings evolved into more open cyclic structures, which indicated the dynamic character of the formed gels. It was observed that signals located near 958 cm^−1^ (band B), 1043 cm^−1^ (band C), and 1144 cm^−1^ (band E) according to SiO_4_ tetrahedrons in Q^2−4^ units, respectively, are more intense for each gel compared to the initial sols. This effect clearly confirms that the polycondensation reaction was in progress. Additionally, the greater intensity of band E (1144 cm^−1^) and band G (1203 cm^−1^) could also be explained by an enhancement of the C–F vibration contribution due to sol-gel host densification. Similar conclusions about polycondensation could be made based on the growing intensity of band F (1186 cm^−1^, LO_4_ mode) and band G (1203 cm^−1^, TO_4_ mode). Moreover, a new deconvoluted component was recorded near 1074 cm^−1^ (band I) for all gels, and it was interpreted as the TO_3_ mode of Si–O–Si siloxane bridges. On the other hand, a band near 1085 cm^−1^ (non-hydrolyzed Si–OC_2_H_5_ groups) was still recorded for SG2 and SG3 gels, although the band was less intense than that of the initial sols. Similarly, as for sols, an infrared signal near 1255 cm^−1^ (band H) according to LO_3_ mode or vibrations of a N–C bond was recorded for SG2 and SG3. The band near 1270 cm^−1^ (band J) corresponding to –CH_2_CH_3_ groups disappeared, which resulted from the partial evaporation of ethyl alcohol from the gels.

During drying of the gels at 35 °C for the next six weeks, xerogels were successfully obtained. This transformation step was correlated with the gradual evaporation of ethyl alcohol and water molecules; hence, a significant decrease in the intensity of the broad infrared band at frequencies >3000 cm^−1^ was observed. After three or four weeks post-synthesis, the identified weight losses gradually decreased until they finally reached almost constant values after seven weeks (16.66%, 29.17%, and 31.03% of the initial masses of SG1, SG2, and SG3, respectively). An interesting weight behavior was observed in the time interval between the fourth and the seventh week post-synthesis. For SG1, negligible differences were noted in the remaining mass (17.00%, 16.80%, 16.67% to 16.66% of the initial mass in the fourth, fifth, sixth, and seventh week, respectively). However, for SG2 and SG3, noticeably larger differences in the remained mass were noted between the fourth and the seventh week post- synthesis (33.87%, 31.50%, 29.52% to 28.38% (SG2) and 36.64%, 34.00%, 31.61% to 29.76% (SG3) in the following weeks). This behavior of the SG2 and SG3 samples could be explained by more efficient trapping of ethyl alcohol and water molecules inside the formed silicate porous structure due to hydrogen bonding with unreacted Si–OH groups (blocked from further polycondensation reaction by DMF additive) or by DMF molecules. In the case of the SG1 sample without the DMF additive, Si–OH groups relatively quickly undergo the polycondensation reaction; thus, water and ethanol can evaporate more easily. It was also observed that during gels → xerogels evolution, the signals originating from C–H bonds recorded at 1390 cm^−1^, 1460 cm^−1^, 2898 cm^−1^, 2910 cm^−1^, and 2980 cm^−1^ were gradually weakened. However, these infrared signals are characterized by greater intensities for SG2 and SG3 during each step of sol-gel transformation, which results from the presence of DMF molecules trapped inside a porous silicate network. Furthermore, for SG2 and SG3 xerogel samples, the weak signal originating from C–N bond vibrations was recorded near the 2812 cm^−1^ frequency region. Moreover, a gradual weakening of the signal located near 1650 cm^−1^ (for SG1 sample) was detected, as well as at 1648 cm^−1^ (for SG2 and SG3 samples), which indicated less participation of C=O vibrations inside the formed sol-gel network.

The deconvolution of the recorded spectra within the 850 cm^−1^–1300 cm^−1^ frequency range revealed that the signal at 878 cm^−1^ (band A) totally disappeared for all fabricated xerogels, suggesting that the two-fold silicate rings wholly transformed into more open cyclic structures. Indeed, the signals according to vibrations inside the four-fold (560 cm^−1^) as well as the six-fold silicate rings (616 cm^−1^) were identified in the recorded IR spectra. The band at 958 cm^−1^ (band B) assigned to SiO_4_ tetrahedrons in Q^2^ units was recorded for each prepared xerogel, and it was observed that the band had the greatest intensity three weeks post-synthesis for SG1 and SG2, and two weeks post-synthesis for SG3. Hence, we could assume that at the early stages of gels → xerogels evolution, the catalyst agent plays a major role in the formation of the silicate sol-gel network, and the addition of nitric acid promotes the polycondensation reaction. In the later stages of this evolution, the intensity of the infrared signal gradually decreased, suggesting that Q^2^ units successfully transformed into Q^3^ and Q^4^ species. Simultaneously, it was reported that the intensity of the 1043 cm^−1^ signal (band C) slightly decreased in the following stages of gels → xerogels evolution, which clearly points to Q^3^ → Q^4^ transformation. However, it should be noted that the band assigned to Q^4^ units (1144 cm^−1^, band E) is better shaped for the SG1 sample compared to the SG2 and SG3 samples. Indeed, since DMF is able to interact with Si–OH groups via hydrogen bonding, they could be efficiently blocked during further polycondensation. Moreover, for all fabricated xerogels, a deconvoluted signal near the 1198 cm^−1^ frequency region (band G; TO_4_ mode of Si-O-Si siloxane bridges and C-F bond vibrations) was seen, whose significant increase was observed for SG1 and SG2 samples, while the increase in the intensity of the signal for the SG3 sample is relatively small. Nonetheless, it should be noted that for the SG3 sample, a presence of LO_4_ modes of Si–O–Si bridges was identified (1186 cm^−1^, band F). Similarly, as for SG2 and SG3 gels, densification of the silicate network of SG2 and SG3 xerogels was confirmed by the deconvoluted component at 1255 cm^−1^ (band H, LO_3_ mode of Si–O–Si bridges). For all fabricated xerogels, deconvolution revealed a very intense component located near the 1067 cm^−1^ frequency region (band I) assigned to the TO_3_ mode of Si–O–Si siloxane bridges. It is quite interesting that the maximum of the signal shifted from 1074 cm^−1^ for gels to a lower wavenumber of 1067 cm^−1^ for xerogels. According to the literature, such a shift could suggest that the xerogels’ silicate structure should be more porous compared to gels [60,64]. The inference about the porosity of the sol-gel structures based on a shift of the TO_3_ mode is taken from the central force network model. In this model, the TO_3_ frequency is associated with the Si–O stretching force constant and the Si–O–Si siloxane bridge angle. The observed shift of wavenumber from 1074 cm^−1^ (for gels) to 1067 cm^−1^ (for xerogels) is related to the dominance of the Si–O bond’s tensile strain effect, and this could indicate an increase in the porosity of the xerogels.

### 2.2. The Evolution of Xerogels into Glass–Ceramics

Generally, controlled heat treatment of fabricated xerogels is responsible for both the in situ crystallization of the LaF_3_ nanophase from La(CF_3_COO)_3_, and the evolution of the sol-gel network [65]. Structural characterization was performed using X-ray diffraction (XRD) as well as transmission electron microscopy (TEM) and, similarly for the case of sol-gel transformation from initial sols to xerogels, infrared spectroscopy (IR).

The creation of the LaF_3_ crystal phase is realized by controlled nucleation through fluorination via creating the La–F bond (the fluorine anions are produced from the cleavage of the C–F bond during heat treatment at 350 °C). Indeed, the following chemical reactions should be considered during sol-gel synthesis:

(1) La(CH_3_COO)_3_ + 3CF_3_COOH → La(CF_3_COO)_3_ + 3CH_3_COOH,
(2) La(CF_3_COO)_3_ → LaF_3_ + MF_3_ + (CF_3_CO)_2_O + CO_2_ + CO.

**Figure 4:**
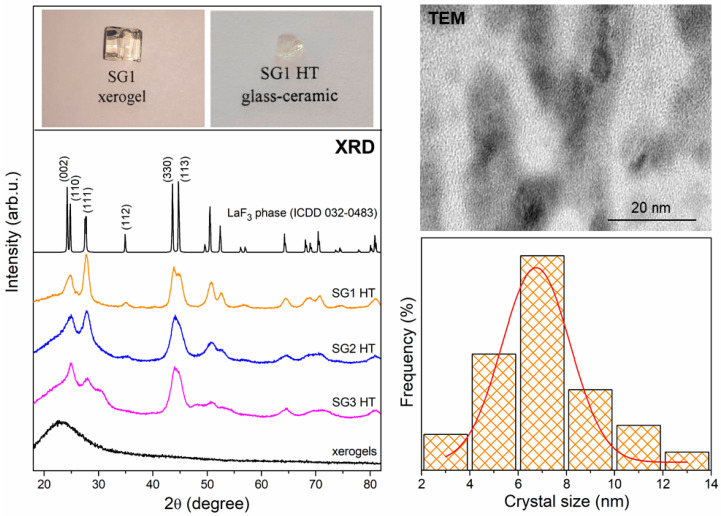
XRD patterns for fabricated xerogels and glass–ceramics. The photographic images of the SG1 xerogel and SG1 HT glass–ceramic, and the TEM image with crystal size distribution for the SG1 HT sample, are also shown.

The first reaction occurs when lanthanum acetate is introduced and mixed with TFA. The second reaction, according to the thermal decomposition of trifluoroacetates such as La(CF_3_COO)_3_, occurs at about 300 °C, which was also confirmed earlier by us based on thermogravimetric analysis (TG) and differential scanning calorimetry (DSC) [50]. Therefore, as demonstrated in Figure 4, a broad halo pattern was recorded for all prepared xerogels, which clearly indicates an amorphous nature without long range structural order. The diffraction reflexes characteristic of the LaF_3_ phase crystallized in the P6_3_/mmc space group (The International Centre for Diffraction Data (ICDD), 032-0483) and were observed after controlled ceramization. The broadening of the recorded diffraction lines indicates the crystallization of the LaF_3_ phase in nanoscale, where the average crystal sizes were estimated to be 8.1 nm (SG1 HT), 7.1 nm (SG2 HT), and 6.8 nm (SG3 HT) from the Scherrer formula [66]:

(1) $$D=\frac{K\lambda }{\beta \cos\theta },$$

where D is the crystal size, K is a constant value (in our calculations, we took K = 1), λ is the X-ray wavelength (1.54056 Å, CuK_α_), β is half of the diffraction line, and θ is the diffraction angle. The results from XRD analysis and TEM imaging of the SG1 HT representative sample (with a histogram for LaF_3_ crystallite size distribution) are presented in Figure 4. The photographic images of the fabricated materials are also shown. The distribution of nanocrystals sizes for SG1 HT ranges from 2 to 14 nm. The crystalline size distribution follows a Gaussian-shaped curve with an average crystalline size of 6–8 nm, and this is well correlated with the results obtained from XRD measurements.

Gorni et al. [32] found that the precipitation of fluoride nanocrystals within a silicate sol-gel host is a fast process that occurs at much lower temperatures than the glass transition temperature, T_g_, of the matrix (~1130 °C). It was also stated that the nanocrystals are unstable in the surrounding silicate host for aging at crystallization. Therefore, the authors observed that after only 1 min of treatment at 550 °C, the fabricated LaF_3_ crystals can be detected with a size of 11 nm, and the further increment of treatment time to 1 h and 20 h led to a decrease in the crystal sizes to 8 nm and 6.5 nm, respectively. These treatments led to a dissolution process favored by the compositional gradient between the host and the crystals. We suppose that these two aspects also play a significant role in crystallization in our xerogels, which could explain the relatively small sizes of the fabricated LaF_3_ nanocrystals. Additionally, the variability in crystal sizes could also be due to the presence of OH groups inside sol-gel hosts; their presence in substantial amounts was verified based on the IR-ATR measurements. In a paper by Almeida et al. [8], a large OH content was found to decrease the thermodynamic barrier for nucleation, and the kinetic barrier for crystal growth resulted in increases in the crystallization rate. Indeed, higher residual OH content tends to decrease the viscosity and increase the nucleation and crystal growth rates. Hence, based on the arguments mentioned above, we could analyze the slight differences in LaF_3_ nanocrystals size for each fabricated glass–ceramic sample: crystals with a larger diameter were identified for the SG1 HT sample (8.1 nm), and slightly smaller yet still comparable crystals were found for SG2 HT (7.1 nm) and SG3 HT (6.8 nm) samples. The former was synthesized with the molar ratio of individual compositions of TEOS:EtOH:H_2_O:CH_3_COOH as 1:4:10:0.5; however, the latter with TEOS:EtOH:DMF: H_2_O:CH_3_COOH(HNO_3_) was 1:2:2:4:0.5(0.4). Thus, the amounts of introduced water and ethyl alcohol were more remarkable for SG1 than for SG2 and SG3. The obtained results may indicate the effect of OH groups originating from residual water and ethyl alcohol on LaF_3_ nanocrystal formation.

**Figure 5:**
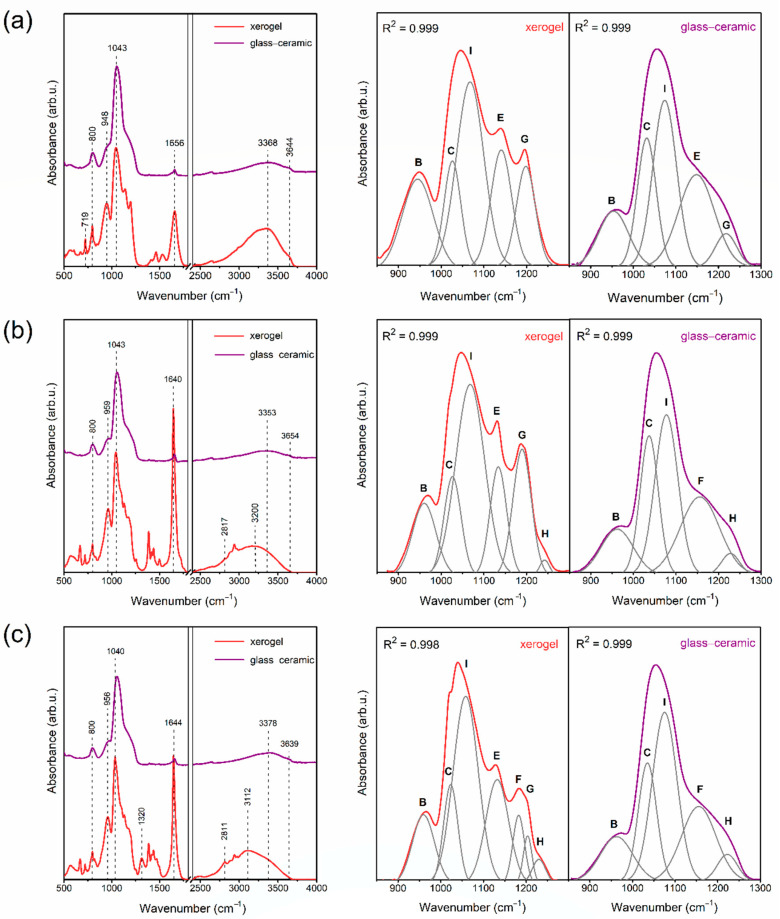
The comparison of IR spectra recorded for SG1 (**a**), SG2 (**b**) and SG3 (**c**) samples before and after controlled heat treatment. Deconvolution within the 850 cm^−1^–1300 cm^−1^ frequency region is also shown.

Besides the crystallization of the LaF_3_ nanophase, another attractive research area involves tracking the structural changes inside the sol-gel host (Figure 5). Since sol-gel systems are dynamic, a rise in temperature from RT up to 350 °C and further heat treatment over the next 10 h induces the evaporation of residual solvents (water and organic liquids) from the microporous structure, as well as the polycondensation reaction with the participation of Q^n^ units. This could be clearly confirmed by the infrared signal behavior at ~1648 cm^−1^ (attributed to vibrations of C=O bond, Si–OH groups and adsorbed water), which is also observable for xerogels (especially for SG2 and SG3); however, it almost completely disappeared for all glass–ceramics. It was also observed that signals within the frequency region of 1300 cm^−1^ to 1500 cm^−1^ disappeared after controlled ceramization due to residual organic solvent evaporation. Moreover, it was reported that a broad band in the frequency region above 3000 cm^−1^ was much more intense for xerogels than for glass–ceramics. For the latter type of sol-gel samples, the band was significantly reduced, which clearly demonstrated the successful evaporation of water and organic liquids and the continuation of the polycondensation reaction.

From the deconvolution of spectra within the 850 cm^−1^–1300 cm^−1^ frequency region, the intensities of the B (958 cm^−1^, Q^2^ units) and C deconvoluted components (1043 cm^−1^, Q^3^ units) decreased due to their conversion into Q^4^ units (band E, 1144 cm^−1^) during progressive polycondensation. However, it should be noted that the intensity of band E after controlled heat treatment also decreased. Nevertheless, such an infrared component could also be assigned to vibrations inside the C–F bond, and when La(CF_3_COO)_3_ undergoes thermal decomposition during controlled ceramization, such vibrations disappear. Therefore, band E originated from vibrations inside Q^4^ units. The change in intensity of band G (1203 cm^−1^) could be explained in a similar way: since the C–F bond is cleaved during the thermal decomposition of La(CF_3_COO)_3_ and further crystallization of the LaF_3_ phase, this component should only originate from the TO_4_ mode of Si–O–Si siloxane bridges. Furthermore, for SG2 and SG3 xerogels, the weak deconvoluted component was observed near 1255 cm^−1^ (band H), which is attributed to vibrations of the N–C bond in DMF molecules, as well as to the LO_3_ mode of Si–O–Si siloxane bridges. This component is also visible after controlled heat treatment of SG2 HT and SG3 HT samples, and it was assigned to vibrations of Si–O–Si. For these samples, the presence of an infrared component near 1186 cm^−1^ (band F), according to LO_4_ mode of Si–O–Si bridges, was also noticed.

### 2.3. Photoluminescence Studies of Prepared Sol-Gel Materials

**Figure 6:**
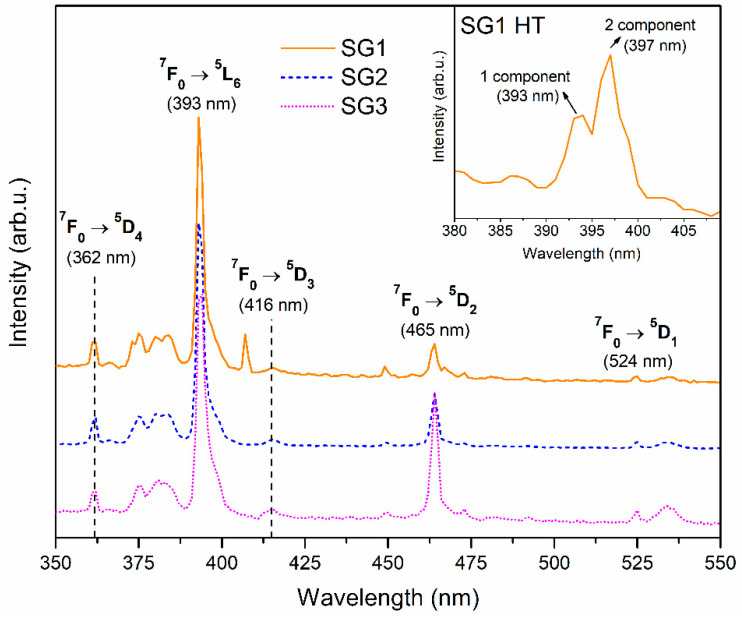
Photoluminescence excitation spectra (PLE) recorded for prepared xerogels monitored for red emission at 612 nm.

The PLE excitation spectra of the fabricated silicate Eu^3+^-doped xerogels are presented in Figure 6. The spectra were recorded within the 350 nm–550 nm UV-Vis irradiation area and monitored for red emission at λ_em_ = 612 nm (the ^5^D_0_ → ^7^F_2_ transition). The recorded narrow bands were assigned to the characteristic intraconfigurational electronic transitions within the 4f^6^ manifold of Eu^3+^ from the ^7^F_0_ ground level into the following upper-lying states: ^5^D_4_ (362 nm), ^5^G_J_, ^5^L_7_ (a set of weak lines in the range from 371 to 390 nm), ^5^L_6_ (393 nm), ^5^D_3_ (416 nm), ^5^D_2_ (465 nm), and ^5^D_1_ (524 nm). Additionally, a weak excitation band corresponding to the ^7^F_1_ → ^5^D_1_ transition was recorded at 535 nm. A well-observed splitting of the ^7^F_0_ → ^5^L_6_ band into two separate components (with maxima located at 393 and 397 nm) for the SG1 HT sample is demonstrated in the inset of Figure 6. Similar results were presented by Yanes et al. [67] for Eu^3+^-doped SiO_2_–LaF_3_ glass–ceramics fabricated at 800 °C. Indeed, the authors proved that the observed splitting is caused by the distribution of Eu^3+^ ions between two different surroundings after controlled heat treatment of the precursor xerogel: the first of them is an amorphous silicate sol-gel host (split component with a maximum at 393 nm), and the second is a LaF_3_ nanocrystal lattice (the prominent component at 397 nm). On this occasion, since such splitting was recorded only for the SG1 HT sample, this could suggest that the migration of the Eu^3+^ ions into a fluoride environment with low phonon energy is strongly promoted when samples are prepared without DMF. However, despite the initial composition of the fabricated sol-gel samples, the ^7^F_0_ → ^5^L_6_ transition maintained the greatest intensity, and therefore the 393 nm near-UV line (397 nm for SG1 HT sample) was used to conduct the emission measurements.

**Figure 7:**
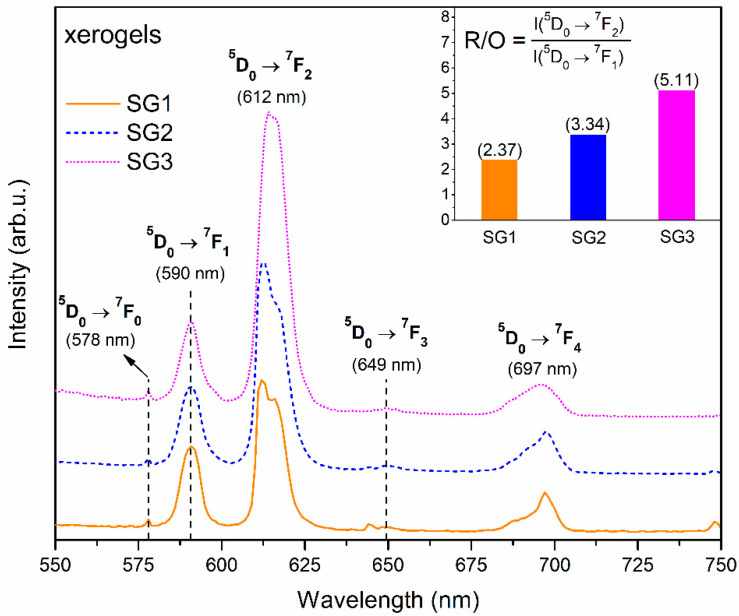
Photoluminescence emission spectra (PL) recorded for fabricated xerogels under near-UV illumination at 393 nm.

Figure 7 presents the PL photoluminescence emission spectra of fabricated silicate xerogels SG1–SG3. The characteristic ^5^D_0_ → ^7^F_J_ luminescence bands of Eu^3+^ ions were detected in the reddish-orange light area and their maxima were located at the following wavelengths: 578 nm (J = 0), 590 nm (J = 1), 612 nm (J = 2), 649 nm (J = 3) and 697 nm (J = 4). It is clearly visible that the ^5^D_0_ → ^7^F_2_ line is the most prominent for all xerogels in the series of recorded bands. Indeed, Eu^3+^ ions are frequently used as spectral probes due to the characteristic nature of their transitions. The ^5^D_0_ → ^7^F_1_ line is a magnetic dipole transition (MD) in nature, for which the intensity is rather independent of the host. In contrast, the ^5^D_0_ → ^7^F_0,2–4_ are electric-dipole transitions (ED) known to be forbidden by the Laporte selection rule, which may occur due to mixing of the 4f orbitals with the opposite parity at low symmetry sites. Among the ED transitions, ^5^D_0_ → ^7^F_2_ has a hypersensitive nature, and its intensity is easily affected by the local vicinity: it is promoted in low symmetric frameworks while being inhibited in more symmetric environments [68]. Hence, we could infer about the symmetry based on the ratio of the integrated intensities of the bands mentioned above, which is well-known in the literature as the R/O ratio (I(^5^D_0_ → ^7^F_2_)/I(^5^D_0_ → ^7^F_1_)). The R/O ratio values for the fabricated xerogels were estimated to be 2.37 (SG1), 3.34 (SG2), and 5.11 (SG3). It is clearly observed that R/O value is strongly dependent on the initial composition of the fabricated sol-gel samples: the smallest value was identified for the SG1 xerogel, a medium value for the SG2 sample, and the highest value for the SG3 xerogel. Hence, such a tendency clearly indicates a growing degree of asymmetry in the immediate vicinity of the Eu^3+^ ions in sol-gel samples in the following direction: SG1 → SG2 → SG3. Indeed, besides H_2_O molecules and CF_3_COO^−^ anions, Eu^3+^ ions are able to coordinate NO_3_^−^ anions and DMF molecules [69]. Thus, the presence of such additional ligands is responsible for disturbing the symmetry in the local framework of Eu^3+^, and thereby the probability of the ^5^D_0_ → ^7^F_2_ transition can be enhanced, causing an observable increase in R/O ratio values.

**Figure 8:**
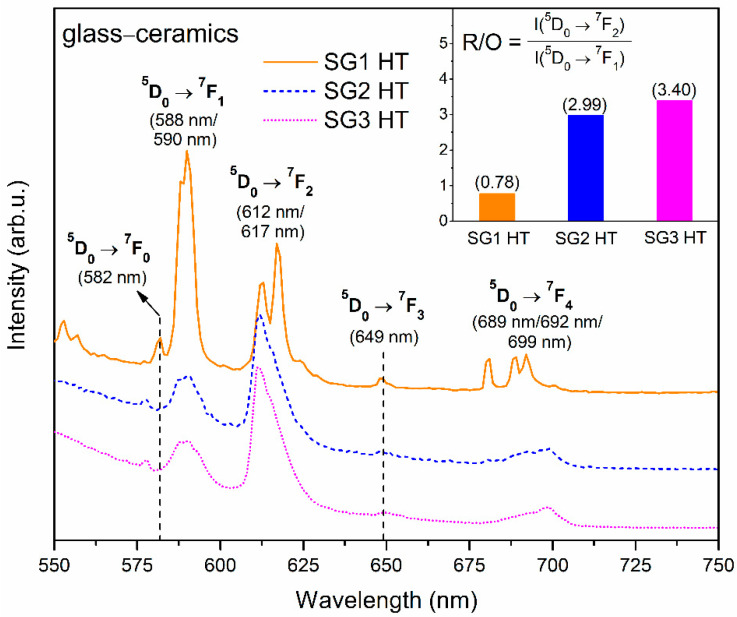
PL spectra recorded for fabricated glass–ceramics recorded under near-UV illumination at 393 nm.

The PL photoluminescence emission spectra recorded for glass–ceramics fabricated during controlled heat treatment at 350 °C are presented in Figure 8. Similarly, for xerogels, characteristic emission bands within the reddish-orange spectral region corresponding to the transitions from the ^5^D_0_ excited level into the ^7^F_J_ lower-lying states were detected. In the case of the SG1 HT sample, the orange ^5^D_0_ → ^7^F_1_ band is dominant, and we denoted a nearly two-fold decline in the R/O ratio value (from 2.37 to 0.78). Moreover, a well-resolved Stark splitting was observed, which points to a crystalline-like environment around the Eu^3+^ ions: the ^5^D_0_ → ^7^F_1_ band was split into two components (588 nm/590 nm), similarly to the ^5^D_0_ → ^7^F_2_ band (612 nm/617 nm), and the ^5^D_0_ → ^7^F_4_ band was split into three components (689 nm/692 nm/699 nm). Such clear splitting results from the partial migration of optically active Eu^3+^ ions into the LaF_3_ precipitated nanocrystal phase. When Eu^3+^ ions are inserted into the crystal lattice, the subsequent energy levels get split by the crystal field effect. The number of sub-levels strongly depends on the symmetry class adjacent to the Eu^3+^ ions, and it is reported in the literature that in LaF_3_ crystal lattices, Eu^3+^ dopant ions occupy C_2v_ symmetry sites [68]. The J term of the ^7^F_J_ levels should split into three (J = 1), five (J = 2), seven (J = 3) and nine (J = 4) sub-levels. Nevertheless, observation of such strong splitting for glass–ceramic systems is quite difficult due to the partial distribution of Eu^3^^+^ ions within the amorphous sol-gel host. A decline in R/O ratio value was also noted for SG2 HT and SG3 HT glass–ceramics (from 3.34 to 2.99 for SG2 HT and from 5.11 to 3.40 for SG3 HT); nevertheless, the reported decrease is not as efficient as for SG1 HT, and the ^5^D_0_ → ^7^F_2_ red emission band is still dominant in the spectra. Besides, no splitting of the recorded emission bands was observed. Thus, based on this collected data, it was established that the most effective migration of Eu^3+^ into the LaF_3_ nanocrystal fraction occurred during the controlled heat treatment of the SG1 xerogel without DMF as an additive during preparation. We suppose that the observed tendency to inhibit Eu^3+^ incorporation into LaF_3_ nanocrystals is caused by the formation of a more stable complex with DMF molecules compared with those formed between H_2_O molecules and CF_3_COO^−^ anions. In this way, we can explain why the large amounts of Eu^3+^ ions in SG2 HT and SG3 HT glass–ceramic samples remained in amorphous sol-gel hosts despite the successful crystallization of the LaF_3_ phase, as was confirmed by XRD and TEM. Moreover, since HNO_3_ is a very strong acid, its addition during sol-gel synthesis could prevent the reaction between Eu(CH_3_COO)_3_ and TFA, which also may inhibit the migration of Eu^3+^ ions into the precipitated LaF_3_ nanocrystals. The very high R/O ratio value calculated for the SG3 HT sample (3.40), which was even greater than for the SG2 HT glass–ceramic (2.99), could suggest that more Eu^3+^ ions remained in the amorphous sol-gel host.

**Figure 9:**
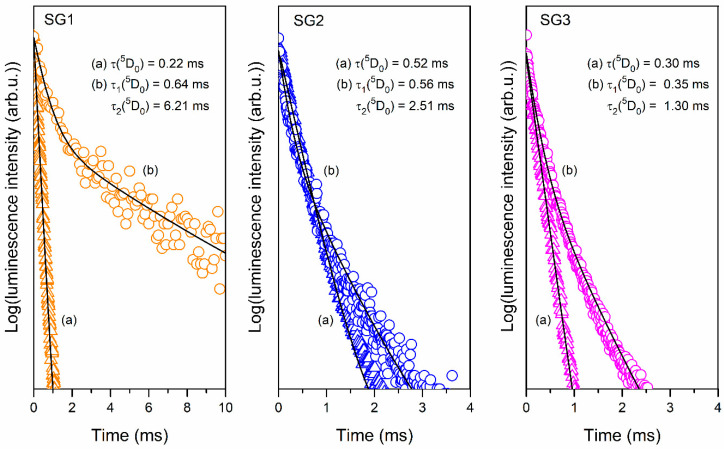
Luminescence decay curves of the ^5^D_0_ state of Eu^3+^ recorded for xerogels (**a**) and glass–ceramics (**b**) (λ_exc_ = 393 nm, λ_em_ = 612 nm).

The further evaluation of the photoluminescent properties of the fabricated sol-gel materials, depending on their initial composition, was based on the decay analysis of the ^5^D_0_ excited state of Eu^3+^ ions (Figure 9). Such decay curves were recorded using near-UV light (^7^F_0_ → ^5^L_6_ transition) as an excitation source, monitoring the red emission (^5^D_0_ → ^7^F_2_ transition). The interpretation of the collected data allows us to establish a clear correlation between the decay profile (mono- or double-exponential) and the type of sol-gel material (i.e., xerogel or glass–ceramic). Indeed, the curves recorded for xerogels are well-fitted to a first exponential decay mode described by the following equation:

(2) $$I\left(t\right)=I_{0}\times \exp\left(-t/\tau \right),$$

where I(t) and I_0_ are the luminescence intensities at time t and t = 0, respectively, while τ is the luminescence decay time. The decay times estimated for xerogels were 0.22 ms (SG1), 0.52 ms (SG2), and 0.30 ms (SG3). In our xerogels, Eu^3+^ are chemically bonded with OH moieties and CF_3_COO^−^ anions (additionally DMF and/or NO_3_^−^ in SG2 and SG3 samples) in complex compounds. It should be noted that high vibrational energies characterize such ligands, i.e., >3000 cm^−1^ (OH groups), ~1660–1140 cm^−1^ (CF_3_COO^−^ anion), ~2810 cm^−1^ (C-N in DMF), and ~1320 cm^−1^ (NO_3_^−^ anion), as was demonstrated in the recorded IR-ATR spectra (Figure 1, Figure 2 and Figure 3). According to the energy gap law, the effective phonons with maximum energy located in the local surroundings of Eu^3+^ ions (*ħω_max_*) generate the most substantial effect on decay times [70,71,72]. In this case, since the vibrational energy of OH moieties is the highest, they play a major role in the non-radiative depopulation of excited states. It should be pointed out that the amounts of water and ethyl alcohol introduced during the synthesis of SG2 and SG3 xerogels (26.9 wt.% and 27.1 wt.%, respectively) were smaller compared to the amounts used during SG1 sample preparation (54.4 wt.%). Hence, smaller amounts of OH moieties adjacent to Eu^3+^ ions favor the radiative depopulation of the ^5^D_0_ excited state, which causes a prolongation of the decay times for SG2 and SG3 xerogels compared with the lifetime estimate for the SG1 sample.

For glass–ceramics, the decay curves are well-fitted to a second exponential decay mode, which can be expressed by the equation:

(3) $$I\left(t\right)/I_{0}=A_{1}\times \exp\left(-t/\tau_{1}\right)+\;A_{2}\times \exp\left(-t/\tau_{2}\right),$$

where A_1_ and A_2_ are amplitudes, while τ_1_ and τ_2_ are the decay times of short and long lifetime components, respectively. The double-exponential decay profile allows us to conclude that the distribution of Eu^3+^ ions between two chemically distinct surroundings is characterized by different phonon energies. In fact, Eu^3+^ ions may migrate during controlled heat treatment into the LaF_3_ crystal lattice, which is formed inside the amorphous sol-gel network as a new chemical environment with low phonon energy (~350 cm^−1^). Due to the low phonon energy of the LaF_3_ lattice, the multi-phonon non-radiative depopulation of the ^5^D_0_ excited state is strongly restricted. However, the remainder of the Eu^3+^ ions are located in an amorphous sol-gel host. According to the IR-ATR spectra recorded for glass–ceramics, it was observed that the intensity of the broad infrared signal originating from OH moieties was significantly reduced; therefore, a major role in non-radiative relaxation is attributed to Q^3^ groups (~1043 cm^−1^). Nevertheless, their phonon energy is greater than that of the LaF_3_ crystal lattice. Such differences in phonon energies in the nearest surroundings of Eu^3+^ ions determine the variable rates of radiative depopulation of the ^5^D_0_ level: in a silicate sol-gel host, the lifetimes are shorter (τ_1_ components), while in the LaF_3_ crystal lattice, the lifetimes are prolonged (τ_2_ components). The distinguished short decay components equaled 0.64 ms (SG1 HT), 0.56 ms (SG2 HT) and 0.35 ms (SG3 HT); meanwhile, the long decay components equaled 6.21 ms (SG1 HT), 2.51 ms (SG2 HT) and 1.30 ms (SG3 HT). From the obtained results, the most extended τ_2_ component was clearly observed for the SG1 HT sample. Since this decay component is strictly related to Eu^3+^ ions located in a low phonon energy fluoride environment, we concluded that differences in τ_2_ luminescence lifetimes for fabricated glass–ceramics could result from the amount of optically active ions entering into the LaF_3_ crystal lattice. Therefore, we assumed an efficient embedding of Eu^3+^ ions into the crystal phase in the SG1 HT sample, and this incorporation efficiency is determined by the types of ligands, which coordinate Eu^3+^ ions in the xerogel host. Based on these obtained results, it was concluded that Eu^3+^ ions form a more stable complex with DMF ligands than with trifluoroacetic anions. Therefore, despite LaF_3_ nanophase crystallization, the migration of Eu^3+^ ions from the silicate sol-gel host was inhibited. In this case, relatively small amounts of Eu^3+^ ions were able to incorporate inside the LaF_3_ crystal lattice; thus, τ_2_ components for SG2 HT and SG3 HT glass–ceramics are relatively short (2.51 ms and 1.30 ms for SG2 HT and SG3 HT, respectively). The lack of DMF ligands in the coordination sphere of Eu^3+^ in the SG1 HT sample is responsible for the efficient migration into fluoride nanocrystals, resulting in long-lived luminescence (τ_2_(^5^D_0_) = 6.21 ms). Simultaneously, it should also be noted that the τ_1_ components for glass–ceramics are slightly elongated compared with the τ values estimated for xerogels. This is due to a significant reduction in the participation of high phonon energy OH groups in the non-radiative deactivation of the ^5^D_0_ excited state in glass–ceramic samples. On the other hand, it should also be noted that only for the SG1 HT sample, an almost three-fold elongation of the τ_1_ component (0.64 ms) compared to the τ before controlled heat treatment (0.22 ms) was reported (for SG2 HT and SG3 HT, the τ_1_ component is comparable with τ). Such elongation of the τ_1_ component for SG1 HT is probably related to the location of the Eu^3+^ ions near fluoride nanocrystals, which may partially reduce the phonon energy in their local surroundings (because they tend to migrate from the silicate sol-gel host into LaF_3_ nanocrystals).

## 3. Materials and Methods

All reagents used during these experiments were from Aldrich Chemical Co. (St. Louis, MO, USA). The solutions of TEOS (98%) and DMF (>98%) in EtOH (98%) and deionized water (from Elix 3 system, Millipore, Molsheim, France) with the addition of acetic (99.5–99.9%) or nitric acid (65%) were introduced into round-bottom flasks. To perform hydrolysis and to initialize a condensation reaction, the components were stirred for 30 min. During the next step, the appropriate amounts of acetates, i.e., La(CH_3_COO)_3_ (99.9%) and Eu(CH_3_COO)_3_ (99.999%) were weighed and dissolved in TFA (TFA, 99%), and the obtained mixtures were added into the TEOS-based solutions. The resultant solutions were mixed for another 60 min. Afterward, the obtained liquid sols were dried at 35 °C for seven weeks to form colorless and transparent solid xerogels (denoted in the text as SG1–SG3). The initial compositions of fabricated sol-gel samples are presented in Table 2.

The further transformation of xerogels into glass–ceramic materials containing LaF_3_ nanocrystals was realized by controlled heat treatment in a muffle furnace (FCF 5 5SHP produced by Czylok, Jastrzębie-Zdrój, Poland) at 350 °C per 10 h (the temperature was raised by 10 °C/min from room temperature). After this procedure, the samples were slowly cooled down to room temperature (denoted in the text as SG1 HT–SG3 HT). To examine the transformation inside the silicate sol-gel hosts, the IR-ATR spectra were recorded on a Nicolet iS50 ATR spectrometer (Thermo Fisher Scientific, Waltham, MA, USA). The formation of LaF_3_ fluoride nanocrystals was verified using X-ray diffraction (XRD, X’Pert Pro diffractometer, Almelo, The Netherlands), and the imaging of nanocrystals was done via high resolution transmission electron microscopy (HR-TEM, JEOL JEM 3010, Tokyo, Japan). The excitation and emission spectra, as well as decay curves, were recorded on a Horiba Jobin Yvon FluoroMax-4 spectrofluorimeter (Horiba Jobin Yvon, Longjumeau, France) supplied with a 150 W Xe lamp. The spectra were recorded with ±0.1 nm resolution, and the decay curves were recorded with ±2 μs accuracy. All structural and luminescence measurements were carried out at room temperature.

## 4. Conclusions

In summary, we studied the impact of initial sols composition (including the addition of DMF and different catalyst agents, i.e., AcOH and HNO_3_) on the structure of oxyfluoride SiO_2_–LaF_3_ nano-glass–ceramics, as well as the distribution of Eu^3+^ ions using several experimental techniques (XRD, TEM microscopy, IR and luminescence measurements). Based on the IR analysis, the influence of the starting sols composition on sol-gel evolution from gels up to nano-glass–ceramics was found. Moreover, it was clearly observed that during gels → xerogels transformation, the water molecules and ethyl alcohol were trapped inside the porous structure due to hydrogen bonding with unreacted Si–OH groups. According to XRD and TEM results, the crystallization of the LaF_3_ phase was confirmed with comparable average crystal size for all fabricated nano-glass–ceramics despite the initial sols composition (8.1 nm, 7.1 nm, and 6.8 nm for SG1–3 HT samples, respectively). From the analysis of R/O ratio values, and changing the luminescence profile of the ^5^D_0_ state from first (xerogels) to second exponential decay (nano-glass-ceramics), it was clearly shown that the lack of DMF additive favors the migration of optically active Eu^3+^ ions into the LaF_3_ crystal phase. Indeed, for the SG1 HT composition (without DMF additive and using AcOH as a catalyst agent), we detected the most extended luminescence lifetime τ_2_ component of 6.21 ms (2.51 ms for SG2 HT, and 1.30 ms for SG3 HT), which predisposes the obtained oxyfluoride nano-glass–ceramic material for its potential use in reddish-orange emitting devices.

## Figures and Tables

**Table 1:** The assignment of individual IR bands in the 500 cm^−1^–4000 cm^−1^ frequency region recorded during all steps of sol-gel transformation. Bands marked with an asterisk (assigned as A–J in the text) were revealed via deconvolution.

IR Signal (cm^−1^)	Assignment
561	four-fold siloxane rings
616	six-fold siloxane rings
719	Si–O bond
800	TO_2_ mode of Si–O–Si siloxane bridges
878 * (A)	two-fold rings, CH_3_CH_2_– groups, C=O groups
958 * (B)	SiO_4_ tetrahedrons in Q^2^ units
1043 * (C)	SiO_4_ tetrahedrons in Q^3^ units
1065, 1074 * (I)	TO_3_ mode of Si–O–Si siloxane bridges
1085 * (D)	non-hydrolyzed Si–O–C_2_H_5_ groups
1144 * (E)	SiO_4_ tetrahedrons in Q^4^ units, C–F bond
1186 * (F)	LO_4_ mode of Si–O–Si siloxane bridges
1198, 1203 * (G)	TO_4_ mode of Si–O–Si siloxane bridges, C–F bond
1255 * (H)	N–C bond, LO_3_ mode of Si–O–Si siloxane bridges
1274 * (J)	CH_3_CH_2_– groups from ethyl alcohol
1320	NO_3_^−^ anion
1390, 1460, 2898, 2910, 2980	C–H bond
1648	C=O groups, Si–OH groups, OH groups (from adsorbed water molecules)
1712	C=O groups
2812	C–N stretching
3230	hydrogen bonded OH moieties from residual water and organic compounds
3398	hydrogen bonded Si–OH groups
3668	non-hydrogen bonded surface Si–OH groups

**Table 2:** The components of fabricated Eu^3+^-doped sol-gel materials.

Sample	Composition (in Molar Ratio)
SG1	TEOS:EtOH:H_2_O:AcOH = 1:4:10:0.5 (90 wt.%)
	TFA:La(CH_3_COO)_3_:Eu(CH_3_COO)_3_ = 5:1:0.05 (10 wt.%)
SG2	TEOS:EtOH:DMF:H_2_O:AcOH = 1:2:2:4:0.5 (90 wt.%)
	TFA:La(CH_3_COO)_3_:Eu(CH_3_COO)_3_ = 5:1:0.05 (10 wt.%)
SG3	TEOS:EtOH:DMF:H_2_O:HNO_3_ = 1:2:2:4:0.4 (90 wt.%)
	TFA:La(CH_3_COO)_3_:Eu(CH_3_COO)_3_ = 5:1:0.05 (10 wt.%)

## Data Availability

The data presented in this study are available on request from the corresponding author.

## Abbreviations

TEOS	tetraethoxysilane
DMF	N,N-dimethylformamide
EtOH	ethyl alcohol
AcOH	acetic acid
TFA	trifluoroacetic acid
PLE	photoluminescence excitation spectra
PL	photoluminescence spectra
